# Relationship between the radiation doses at nonenhanced CT studies using different tube voltages and automatic tube current modulation during anthropomorphic phantoms of young children

**DOI:** 10.1002/acm2.12192

**Published:** 2017-10-05

**Authors:** Takanori Masuda, Yoshinori Funama, Masao Kiguchi, Kazuaki Osawa, Syouichi Suzuki, Takayuki Oku, Koichi Sugisawa, Tomokazu Shouji, Kazuo Awai

**Affiliations:** ^1^ Department of Radiological Technology Tsuchiya General Hospital Naka‐ku Japan; ^2^ Department of Medical Physics Faculty of Life Sciences Kumamoto University Kumamoto Japan; ^3^ Department of Diagnostic Radiology Graduate School of Biomedical Sciences Hiroshima University Hiroshima Japan; ^4^ Department of Radiological Technology Saiseikai Chuwa Hospital Nara Japan; ^5^ Department of Diagnostic Radiology Fujita Health University School of Health Science Aichi Japan; ^6^ Department of Diagnostic Radiology Keio University School Tokyo Japan; ^7^ Department of Radiology Jikei University Kashiwa Hospital Chiba Japan

**Keywords:** low tube voltage, pediatric computed tomography, radiation dose, tube current modulation

## Abstract

To compare the radiation dose and image noise of nonenhanced CT scans performed at 80, 100, and 120 kVp with tube current modulation (TCM) we used anthropomorphic phantoms of newborn, 1‐year‐old, and 5‐year‐old children. The noise index was set at 12. The image noise in the center of the phantoms at the level of the chest and abdomen was measured within a circumscribed region of interest. We measured the doses in individual tissues or organs with radio‐photoluminescence glass dosimeters for each phantom. Various tissues or organs were assigned and the radiation dose was calculated based on the international commission on radiological protection definition. With TCM the respective radiation dose at tube voltages of 80, 100, and 120 was 29.71, 31.60, and 33.79 mGy for the newborn, 32.00, 36.79, and 39.48 mGy for the 1‐year‐old, and 32.78, 38.11, and 40.85 mGy for the 5‐year‐old phantom. There were no significant differences in the radiation dose among the tube voltages and phantoms (*P* > 0.05). Our comparison of the radiation dose using anthropomorphic phantoms of young children showed that the radiation dose of nonenhanced CT performed at different tube voltages with TCM was not significantly different.

## 1 INTRODUCTION

Computed tomography (CT) is a valuable imaging technique that helps to diagnose and manage some pediatric medical conditions.^1^, ^2^, ^3^ However, CT can result in a high amount of cumulative radiation.^4^ As children can be expected to have a longer life expectancy than older individuals, their potential risk for developing cancer due to radiation exposure from diagnostic imaging is also higher.^5^, ^6^ Special attention must, therefore, be paid to pediatric CT scans because their life expectancy is longer than that of adults.

By using low tube voltage scans, decreasing the tube voltage increases contrast resolution, and the image noise may be higher at the same dose level.^7^, ^8^, ^9^ For CT angiography studies, iodinated contrast material is used to improve enhancement without a substantial increase in the image noise.^9^ Kalender et al.^10^ recommended that lower tube voltages be applied at contrast‐enhanced pediatric CT. However, there are few reports on the radiation dose in pediatric unenhanced CT studies using lower tube voltages.

We used anthropomorphic phantoms of young children to evaluate the radiation dose for unenhanced pediatric scans performed at different tube voltages with tube current modulation (TCM).

## 2 MATERIALS AND METHODS

### 2.A Phantoms

**Figure 1:**
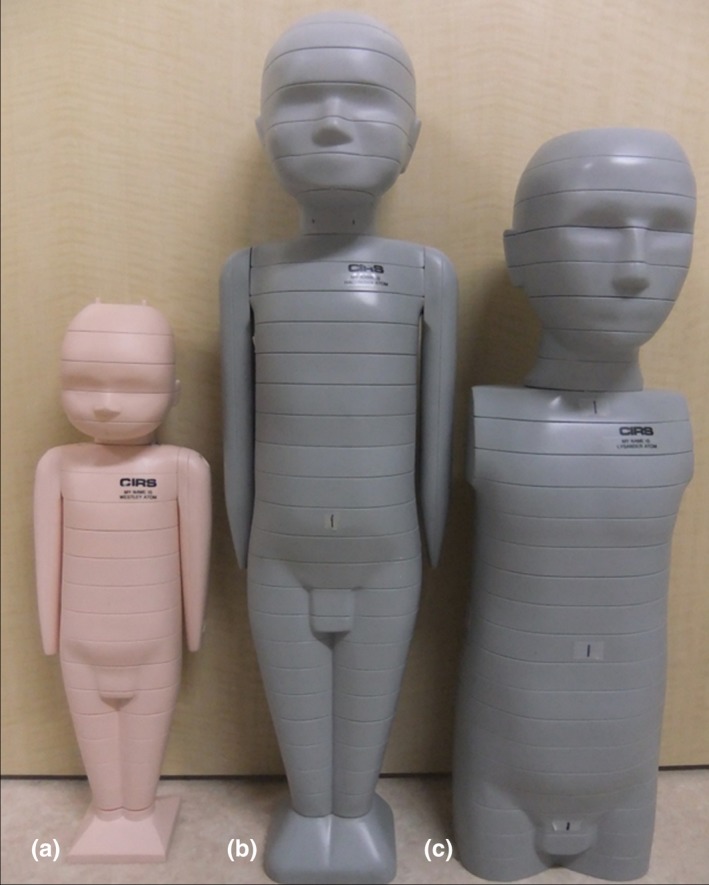
Anthropomorphic phantoms of a newborn (left), a 1‐year‐old (center), and a 5‐year‐old human (right).

**Figure 2:**
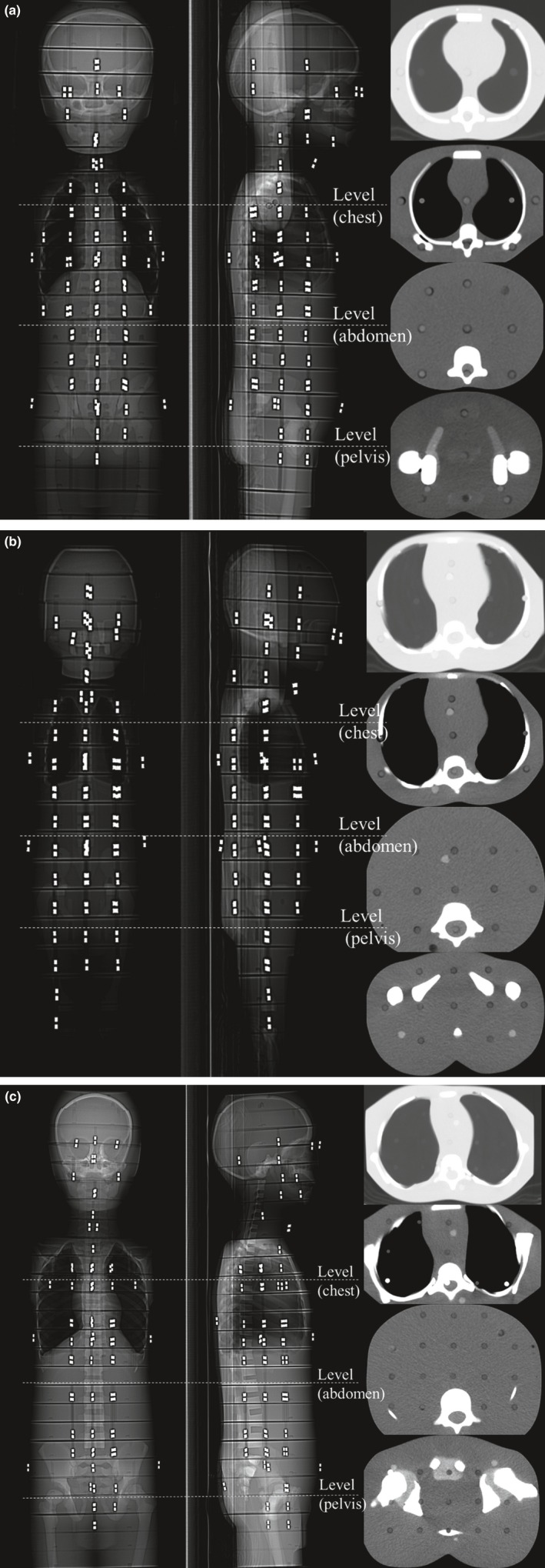
(a) Anthropomorphic phantoms of a newborn for scout views and axial views at chest, abdomen, and pelvis level. (b) Anthropomorphic phantoms of a 1 year‐old for scout views and axial views at chest, abdomen, and pelvis level. (c) Anthropomorphic phantoms of a 5‐year old for scout views and axial views at chest, abdomen, and pelvis level.

We used three pediatric anthropomorphic phantoms (ATOM Phantom, CIRS, Rev2, Q2 Norfolk, Virginia, USA) that represented an average newborn, a 1‐year‐old, and a 5‐year‐old child (Figs. 1 and 2). Phantoms from this manufacturer feature holes at the site of 22 radiobiologically important organs; the sites are optimized to facilitate precise calculations using the minimum number of necessary detectors. The assumed body weight (BW) and body height (BH) for the newborn and the 1‐year old and 5‐year old were 3.5 kg and 51.0 cm, 10.0 kg and 75.0 cm, and 19.0 kg and 110.0 cm, respectively. The phantoms were made of radiologically equivalent tissue materials with internal structures that included artificial skeletons, lungs, and soft tissues formulated for the accurate simulation of clinical exposures.

### 2.B CT Scanning

**Figure 3:**
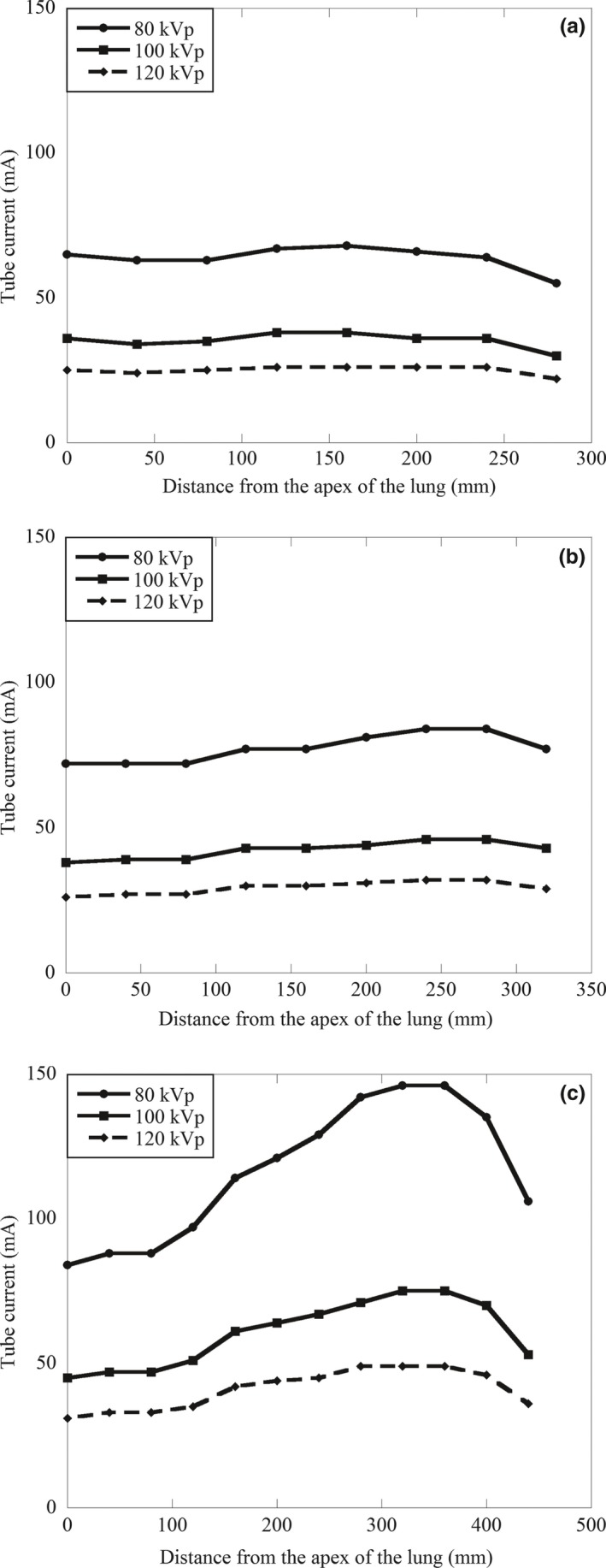
(a) The mA profile along the z‐axis from the thorax to the lower abdomen using anthropomorphic phantom of a newborn. (b) The mA profile along the z‐axis from the thorax to the lower abdomen using anthropomorphic phantom of a 1 year‐old. (c) The mA profile along the z‐axis from the thorax to the lower abdomen using anthropomorphic phantom of a 5‐year old.

All CT scans were performed on a 64 detector row scanner (Lightspeed VCT; GE Healthcare, Milwaukee, WI) from the thorax to the lower abdomen including the entire lungs. The scan range was 265 mm for the newborn, 300 mm for the 1‐year‐old, and 405 mm for the 5‐year‐old phantom. The scanning parameters were helical mode, beam width 40.0 mm, section thickness 5.0 mm, pitch factor 0.984 mm/rotation, gantry rotation time 0.4 sec, 64 × 0.625 mm detector collimation, settings for the small scan field of view were 100, 150, and 150 mm for the newborn, the 1‐year‐old, and the 5‐year‐old phantom, respectively, matrix size 512 × 512, reconstructed mode for plus mode, standard reconstruction kernel. The applied tube voltages were 80, 100, and 120 kVp, and the tube current was changed from 20 to 330 mA to maintain the image quality (noise index at 12) using automatic tube current modulation for all phantoms (Figs. 3a–3c).^11^ We verified that none of the scans reached the maximum mA. CT images were acquired with filtered back projection (FBP) algorithms under the standard kernel/filter.

### 2.C Image noise and dose‐length product at different tube voltages

At each tube voltage and in all phantoms we measured the image noise [standard deviation (SD) of the CT number] in the center of the phantom at the level of the chest and abdomen within a circumscribed 6.0 mm diameter region of interest (ROI). For each scan we acquired 100 consecutive images in the z direction using a CT workstation (Advantage Windows 4.4, GE Healthcare) to separate the 100 images from the upper chest to below the pelvis. The slice thickness was 5.0 mm for FBP algorithms using standard kernel/filter. The mean value for the SD of the CT number was calculated. Dose‐length product (DLP) values displayed on the CT console were recorded.

### 2.D Dosimeters

**Figure 4:**
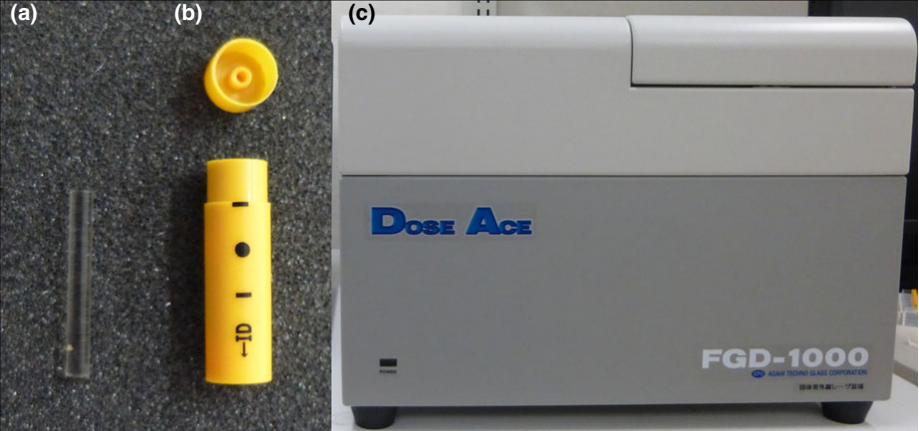
GD352M glass dosimeters (left), a tin filter (center), and a FDG 1000 reader (right).

Glass dosimeters are accumulation‐type, solid‐state dosimeters.^12^ They take advantage of the radio‐photoluminescence of silver‐activated phosphate glass and are comprised of rod‐shaped silver‐activated phosphate glass, a plastic capsule, and an automatic reader unit. Their weight composition is P (31.55%), O (51.16%), Al (6.12%), Na (11.0%), and Ag (0.17%) and their effective atomic number and density are 12.039 and 2.61 g/cm^2^, respectively. Measurable doses range from 10 μGy to 10 Gy in standard mode and from 1 to 500 Gy in high‐dose mode. We used GD352M glass dosimeters (Asahi Techno Glass, Japan) designed for low‐energy photon beams. A tin filter (Dose Ace, glass dosimeter; Asahi Techno Glass, Shizuoka, Japan) compensates for the high response of low‐energy photons (Fig. 4). The readout area for high‐dose range is located at between 0.4 and 1 mm, a total length of 6 mm and a total volume of 0.47 mm^3^, from the nonseries end in the readout area; while the low‐dose range is located from 1 to 6 mm with a volume of 0.47 mm^3^.^13^, ^14^

### 2.E Dose measurements

To obtain clinically suitable dose values the positioning of the phantoms was in the center to mimick the position of a patient undergoing the treatment of a particular anatomical region. For head and neck scans, the phantom was aligned with the center of the philtrum at the isocenter of the beam. The irradiated region during CT included a part of the esophagus, the thyroid, salivary glands, oral mucosa, extrathoracic region, and brain. For chest scans, the beam isocenter was placed in the center of the body at an axial plane near the center of the lungs. The irradiated region for chest scans covered the lungs, part of the esophagus, the breast, liver, thymus, heart, spleen, adrenals, pancreas, gall bladder, kidneys, part of the small intestine, and the stomach. For pelvis scans, the center of the sacrum was placed at the beam isocenter. The irradiated region in the pelvis scan consisted of the colon, ovaries, small intestine, cervix, and bladder. To ensure reproducibility, cross hairs at the anterior and the lateral side were marked and always aligned with the cross line of the alignment lights.

**Figure 5:**
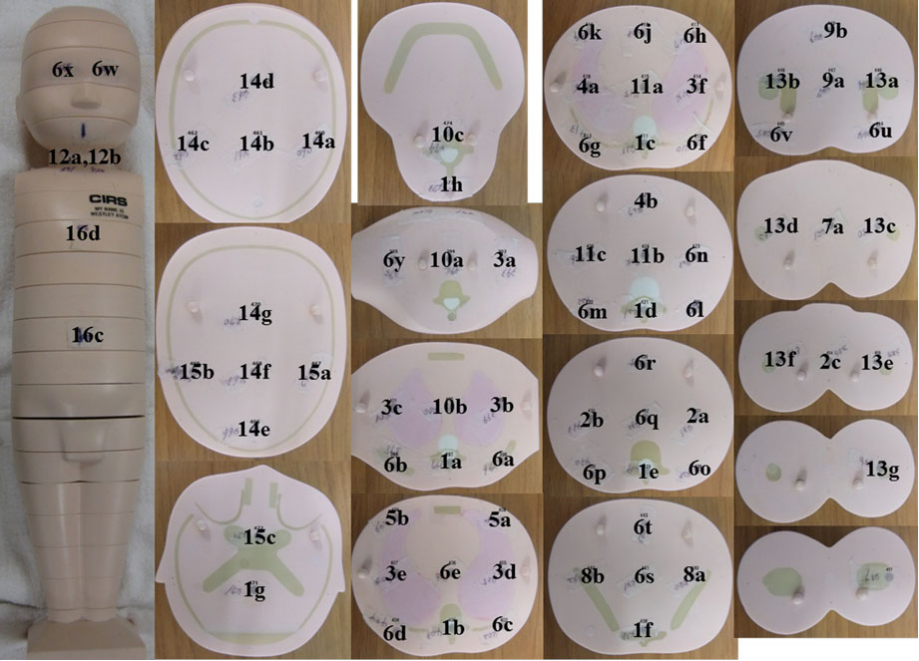
The portions measured with radio‐photoluminescence glass dosimeters in the anthropomorphic newborn phantom.

**Figure 6:**
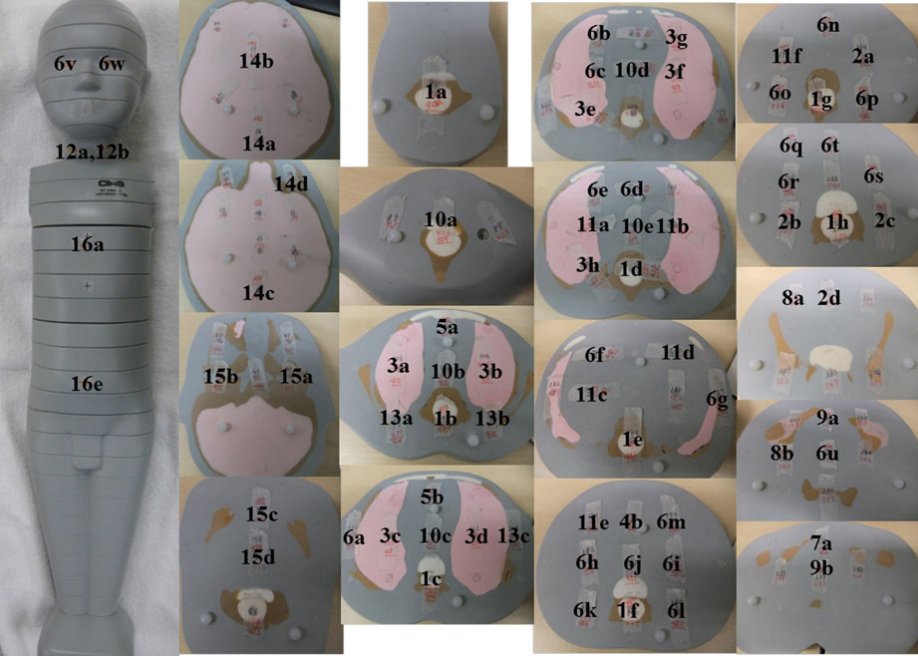
The portions measured with radio‐photoluminescence glass dosimeters in the anthropomorphic 1‐year old.

**Figure 7:**
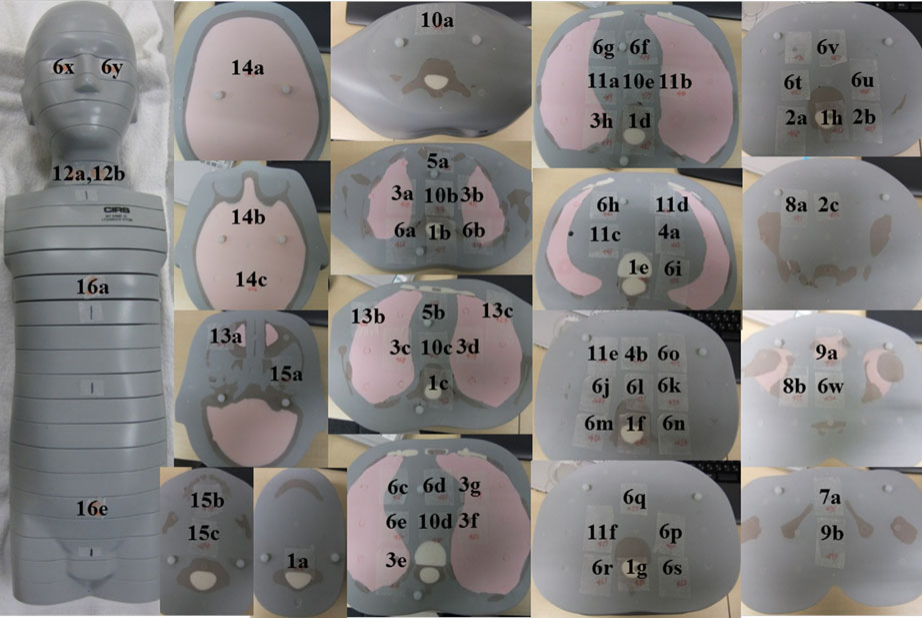
The portions measured with radio‐photoluminescence glass dosimeters in the anthropomorphic 5‐year old.

We measured the individual doses in the tissues or organs of the 3 phantoms based on the definitions of the international commission on radiological protection (ICRP). Radio‐photoluminescence glass dosimeters with a tin filter were inserted at the position of the individual organs, at the center and at the front, back, left, and right sides of the phantom surface (Figs. 5, 6, 7). We took 83 measurements in the newborn, 83 in the 1‐year‐old, and 83 in the 5‐year‐old phantoms. Dose measurements were performed on an FDG 1000 reader (ATGC 2004; Asahi Techno Glass) (Fig. 4).

### 2.F Comparison of the radiation dose between the 10 cm ionization chamber, computed tomography dose index volume, and radio‐photoluminescence glass dosimeters

We compared the radiation dose between the 10 cm ionization chamber and computed tomography dose index volume (CTDIvol) of the console displayed dose using CT equipment because we had to check the accuracy of the CTDIvoi of the console displayed dose. CTDIvol was good linearity of reference dose with the ionization chamber. We also compared the radiation dose between the 10 cm ionization chamber and radio‐photoluminescence glass dosimeters using X‐ray of general radiographic equipment due to the need to check the radio‐photoluminescence glass dosimeters traceability. The radio‐photoluminescence glass dosimeters were good for linearity of reference dose with the ionization chamber.

### 2.G Size‐specific dose estimate calculations

Size‐Specific Dose Estimates (SSDE) were measured using the American Association of Physicists in Medicine (AAPM) Report 204—that is, to use of the anteroposterior (AP) parameter as a measurement of body thickness from anterior to posterior and the lateral (LAT) parameter as a measurement of the body thickness from left to right and the summation of the AP and lateral dimensions and multiply the respective conversion factors with the CTDIvol at 16 cm.

### 2.H Evaluation of organ and effective doses

Various tissues or organs were assigned an effective dose (ED) based on the definitions of the international commission on radiological protection (ICRP) by using three pediatric anthropomorphic phantoms. The ED was defined as follows:

$$ED=absorbeddose\times W_{R}\times W_{T},$$

where W_T_ was the tissue‐weighing factor and W_R_ was the radiation weighting factor for both ICRP 60 and ICRP 103 definitions. For X‐ray photons, W_R_ = 1 and the absorbed doses were the measurement values of the RPLGD.

### 2.I Reducing radiation dose when noise is to be matched

To clarify the relative radiation dose required at each tube voltage to obtain the same noise level for all three phantoms, we plotted the all data points for each corresponding dose value (CTDIvol, SSDE, effective dose, and each organs) matching the noise and scale their dose values for each category (new born chest, new born abdomen, 1‐year‐old chest, 1‐year‐old abdomen, 5‐year‐old chest, and 5‐year‐old abdomen).

### 2.J Statistical analysis

For the analysis, measurement of radiation dose in each Glass dosimeter at the different tube voltages with TCM we used Kruskal‐Wallis analysis of variance. When the assumption of homogeneity of variances was not verified, we performed Steel‐Dwass analysis. Values of *P* < 0.05 were considered to be statistically significant. Statistical analyses were with the free statistical software “R” (R,version 3.2.2; The R Project for Statistical Computing; http://www.r-project.org/).

## 3 RESULTS

### 3.A CTDIvol and DLP displayed on the CT console, size‐specific dose estimate, and effective dose

CTDIvol displayed on the CT console, DLP displayed on the CT console for chest and abdomen, SSDE for chest and abdomen, and ED showed in Table 1.

**Table 1:** Computed tomography dose index and dose‐length product values displayed on the CT console and size‐specific dose estimate

	80 kVp				100 kVp				120 kVp			
	CTDIvol	DLP	SSDE	ED	CTDIvol	DLP	SSDE	ED	CTDIvol	DLP	SSDE	ED
	(mGy)	(mGy·cm)	(mGy)	(mSV)	(mGy)	(mGy·cm)	(mGy)	(mSV)	(mGy)	(mGycm)	(mGy)	(mSV)
New born												
Chest	1.81	22.6	2.37	2.30	1.81	22.6	2.37	2.50	1.95	24.3	2.55	2.60
Abdomen		33.9	2.37			33.9	2.37			36.5	2.55	
1 year‐old												
Chest	2.19	30.3	2.56	2.40	2.15	29.8	2.51	2.90	2.29	31.7	2.68	3.20
Abdomen		45.5	2.52			44.7	2.48			47.5	2.63	
5 year‐old												
Chest	3.33	60.1	3.39	2.60	3.14	56.6	3.2	2.90	3.25	58.7	3.32	3.20
Abdomen		90.1	3.33			84.9	3.14			88.1	3.25	

### 3.B Radiation doses of whole‐body exposure

**Figure 8:**
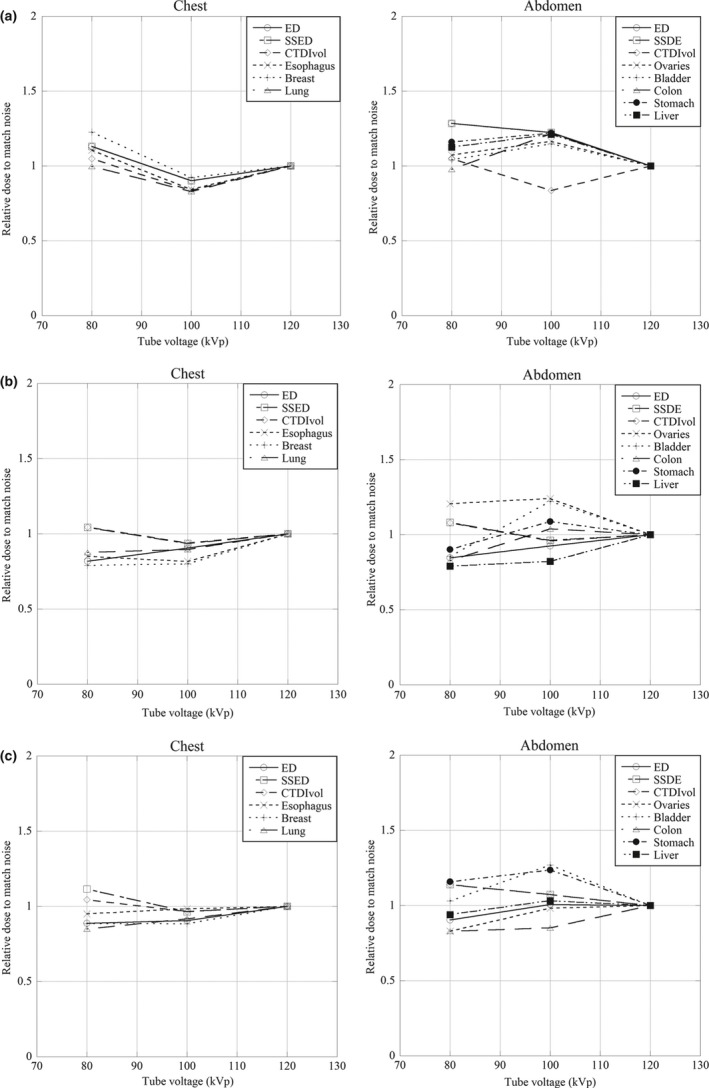
(a) Graph shows the relative radiation dose required at each tube potential to obtain the same noise level for chest and abdomen of anthropomorphic newborn phantom. (b) Graph shows the relative radiation dose required at each tube potential to obtain the same noise level for chest and abdomen of anthropomorphic 1‐year‐old phantom. (c) Graph shows the relative radiation dose required at each tube potential to obtain the same noise level for chest and abdomen of anthropomorphic 5‐year old.

In Tables 2, 3, 4, we present the radiation dose for each tissue or organ of the three phantoms; the noise index was 12 and TCM was applied for scans at the different voltages. There was no significant difference in the radiation dose among the tube voltages (*P* > 0.05). However, in all phantoms, the radiation dose was minimally decreased at the lower tube voltages (80, 100 kVp). Figure 8 showed that if the noise level in an image obtained at 120 kVp was to be matched, the possibility of dose reduction at 80 kVp were 0.1%, 22.6%, 10.6%, 0.1%, 13.1%, 11.3% in newborn chest and 14.9%, 11.5%, 4.9%, 4.4%, 11.5%, 11.3% in 5‐year‐old chest for lung, breast, esophagus, CTDIvol, SSDE, ED, respectively. With respect to abdomen, the possibility of dose reduction at 80 kVp were 12.6%, 15.9%,3.4%, 4.1%,7.2%, 4.9%, 27.6%, 27.4% in newborn abdomen and 6.1%, 15.8%, 16.9%, 3.1%, 16.5%, 13.9%, 13.8%, 9.6% in 5‐year‐old abdomen for liver, stomach, colon, bladder, ovaries, CTDIvol, SSDE, ED, respectively. In newborn abdomen, 1‐year‐old abdomen, and 5‐year‐old abdomen for relative dose to match noise were higher than that of chest in lower tube voltage. Likewise, relative dose to match noise of 80 kVp was higher than 100 kVp in all anthropomorphic phantoms.

**Table 2:** Radiation dose calculation with SD‐based TCM (SD 12) during chest and abdominal CT of a newborn anthropomorphic phantom

Tissue or organ	Measurement portion	Number of dosimeters	Measured dose (mGy)		
			80 kVp	100 kVp	120 kVp
Bone marrow	1	8	1.39 ± 0.19	1.44 ± 0.23	1.65 ± 0.27
Colon	2	3	2.10 ± 0.59	2.74 ± 0.37	2.76 ± 0.17
Lung	3	6	2.24 ± 0.26	2.25 ± 0.20	2.54 ± 0.37
Stomach	4	2	2.70 ± 0.02	3.19 ± 0.56	2.99 ± 0.32
Breast	5	2	2.69 ± 0.21	2.54 ± 0.23	2.48 ± 0.28
Remainder tissues	6	24	2.23 ± 0.29	2.32 ± 0.23	2.50 ± 0.20
Testes	7	1	2.59 ± 0.00	2.83 ± 0.00	2.79 ± 0.00
Ovaries	8	2	2.18 ± 0.21	2.48 ± 0.23	2.61 ± 0.48
Bladder	9	2	2.18 ± 0.52	2.52 ± 0.20	2.69 ± 0.59
Esophagus	10	3	2.30 ± 0.43	2.20 ± 0.22	2.35 ± 0.49
Liver	11	3	2.55 ± 0.17	2.88 ± 0.43	2.91 ± 0.26
Thyroid	12	2	2.82 ± 0.07	2.66 ± 0.08	3.19 ± 0.54
Bone surface	13	7	1.44 ± 0.23	1.46 ± 0.25	1.62 ± 0.31
Brain	14	7	0.14 ± 0.11	0.15 ± 0.05	0.18 ± 0.08
Salivary glands	15	3	0.23 ± 0.13	0.25 ± 0.11	0.40 ± 0.13
Skin	16	8	2.36 ± 0.02	2.36 ± 0.02	2.83 ± 0.02

The measured portions are identified in Fig. 5.

**Table 3:** Radiation dose calculation with SD‐based TCM (SD 12) during chest and abdominal CT of a 1‐year‐old anthropomorphic phantom

Tissue or organ	Measurement portion	Number of dosimeters	Measured dose (mGy)		
			80 kVp	100 kVp	120 kVp
Bone marrow	1	8	1.14 ± 0.25	1.44 ± 0.32	1.52 ± 0.35
Colon	2	4	2.11 ± 0.13	2.93 ± 0.07	2.88 ± 0.16
Lung	3	8	2.31 ± 0.63	2.58 ± 0.30	2.88 ± 0.52
Stomach	4	2	2.82 ± 0.09	3.76 ± 0.84	3.53 ± 0.36
Breast	5	2	2.67 ± 0.69	2.72 ± 0.50	4.86 ± 0.17
Remainder tissues	6	23	2.25 ± 0.26	2.95 ± 0.29	3.07 ± 0.26
Testes	7	1	2.71 ± 0.00	3.27 ± 0.00	3.44 ± 0.00
Ovaries	8	2	2.20 ± 0.76	3.41 ± 0.34	2.06 ± 0.58
Bladder	9	2	2.18 ± 0.36	3.45 ± 0.39	2.88 ± 0.39
Esophagus	10	5	2.35 ± 0.51	2.46 ± 0.35	3.01 ± 0.36
Liver	11	6	2.37 ± 0.39	3.06 ± 0.58	3.80 ± 0.65
Thyroid	12	2	4.34 ± 0.85	2.82 ± 0.93	2.61 ± 0.27
Bone surface	13	3	1.52 ± 0.20	1.60 ± 0.25	1.79 ± 0.29
Brain	14	4	0.19 ± 0.04	0.25 ± 0.05	0.27 ± 0.03
Salivary glands	15	3	0.49 ± 0.20	0.63 ± 0.20	0.81 ± 0.13
Skin	16	8	2.83 ± 0.03	2.83 ± 0.02	2.83 ± 0.01

The measured portions are identified in Fig. 6.

**Table 4:** Radiation dose calculation with SD‐based TCM (SD 12) during chest and abdominal CT of a 5‐year‐old anthropomorphic phantom

Tissue or organ	Measurement portion	Number of dosimeters	Measured dose (mGy)		
			80 kVp	100 kVp	120 kVp
Bone marrow	1	8	1.16 ± 0.44	1.19 ± 0.39	1.70 ± 0.35
Colon	2	3	2.68 ± 0.08	2.75 ± 0.21	3.59 ± 0.80
Lung	3	8	2.41 ± 0.69	2.65 ± 0.99	2.89 ± 0.55
Stomach	4	2	3.73 ± 0.13	3.98 ± 0.69	3.58 ± 0.18
Breast	5	2	2.70 ± 0.71	2.75 ± 0.53	3.11 ± 0.46
Remainder tissues	6	25	2.23 ± 0.43	2.32 ± 0.61	2.92 ± 0.34
Testes	7	1	2.94 ± 0.00	3.72 ± 0.00	4.34 ± 0.00
Ovaries	8	2	1.99 ± 0.12	3.69 ± 0.85	4.18 ± 0.81
Bladder	9	2	2.90 ± 0.06	3.69 ± 0.49	3.13 ± 0.26
Esophagus	10	5	2.74 ± 0.48	2.89 ± 0.48	2.94 ± 0.80
Liver	11	6	3.03 ± 0.71	3.33 ± 0.45	3.59 ± 0.66
Thyroid	12	2	2.43 ± 0.08	4.04 ± 0.31	2.68 ± 0.42
Bone surface	13	3	1.39 ± 0.28	1.35 ± 0.23	2.23 ± 0.41
Brain	14	3	0.21 ± 0.02	0.25 ± 0.01	0.36 ± 0.07
Salivary glands	15	3	0.42 ± 0.22	0.42 ± 0.11	0.60 ± 0.17
Skin	16	8	2.83 ± 0.02	2.83 ± 0.02	3.30 ± 0.02

The measured portions are identified in Fig. 7.

### 3.C Radiation doses for center and periphery organs

The radiation dose for lung was at 80, 100, and 120 kVp was 2.24, 2.25, and 2.54 mGy for the newborn, 2.31, 2.58, and 2.88 mGy for the 1‐year‐old, and 2.41, 2.65, and 2.89 mGy for the 5‐year‐old phantom. The absorbed dose for skin was at 80, 100, and 120 kVp was 2.36, 2.36, and 2.83 mGy for the newborn, 2.83, 2.83, and 160 2.83 mGy for the 1‐year‐old, and 2.41, 2.65, and 2.89 mGy for the 5‐year‐old phantom. There was no significant difference in the absorbed dose among the tube voltages (*P* > 0.05).

### 3.D Image noise variations

Variations in the image noise on nonenhanced scans with TCM and a noise index of 12 are shown in Table 5. With TCM there were variations in the image noise at all applied tube voltages.

**Table 5:** Relationship of the image noise at three tube voltages and the anthropomorphic phantom

	Image noise (HU)		
	80 kVp	100 kVp	120 kVp
Newborn			
Chest	8.4 (6.2–10.5)	7.5 (6.1–11.8)	7.9 (6.4–11.6)
Abdomen	8.5 (6.0–10.7)	8.3 (6.5–10.8)	7.5 (6.0–10.2)
1‐year‐old			
Chest	9.3 (7.1–11.9)	8.9 (7.0–11.8)	8.9 (7.0–11.5)
Abdomen	10.2 (7.3–11.9)	9.7 (7.1–11.8)	9.6 (7.2–11.9)
5‐year‐old			
Chest	10.4 (8.0–12.9)	10.0 (8.1–12.8)	10.3 (8.0–12.3)
Abdomen	11.6 (8.2–13.9)	11.6 (8.4–13.9)	11.0 (8.2–13.9)
		HU, Hounsfield units	

Data are the median standard deviation of the CT number and the minimum and maximum values.

## 4 DISCUSSION

We identified the radiation dose by scanning at 80, 100, and 120 kVp with TCM using anthropomorphic phantoms for a newborn, and a 1‐year‐old, and a 5‐year‐old child. Comparison of the radiation dose showed that at these tube voltages the delivered radiation dose was not significantly different.

Yu, et al.^9^ reported that the noise level in an image obtained at 120 kVp is to be matched, and the potential for dose reduction at lower tube potentials is limited or nonexistent. For the 10 cm phantom, radiation dose is reduced by 12% at 80 kVp and by 8% at 100 kVp compared with the dose at 120 kVp. For the 25 cm phantom, a 29% dose increase is required at 80 kVp to match the noise level at 120 kVp. We used the three types of anthropomorphic phantoms and radio‐photoluminescence glass dosimeters to evaluate how the lower tube voltages affect the dose more clinically. When using the 10 cm phantom, it corresponds to 1‐year‐old anthropomorphic phantom. In our results, the possibility of dose reduction were 12.5%, 20.5%, 14.8%, 20.1%, 9.9%, 17.3%, 14.6%, and 20.1% at 80 kVp and 11.5%, 19.5%, 18.8%, 17.8%, 8.7%, 3.8%, 21.7%, and 23.6% at 100 kVp for lung, breast, esophagus, liver, stomach, colon, bladder, and ovaries, respectively. Compared with their results, our results showed more high value. Even if they were used, a similar result may be obtained in the pediatric area.

Life expectancy of children is longer than that of adults, their CT studies require the accurate evaluation of the whole‐body exposure. The dose parameters routinely displayed on scanner consoles include CTDI_vol_ and DLP. As they are based on measurements in standard CT dose phantoms that are 16 or 32 cm in diameter. The effective dose is thought to correlate best with the overall stochastic radiation risk.^15^ The dose calculations based on CTDI or DLP are readily available indicators of the radiation dose in CT studies. The organ dose and the effective dose can be estimated from the CTDI or the DLP using conversion factors derived from the Monte Carlo simulation of photon interactions within a simplified mathematical model of the human body.^16^ Accordingly, new series of Monte Carlo calculations have been carried out at National Radiological Protection Board for a family of 6 geometric mathematical phantoms, representing ages from newborn to adult. These have formed the basis for the derivation of broad pediatric enhancement factors, which summarize the increased doses to small children relative to those of adults under similar conditions of CT exposure.^17^ However, in children, calculation of the effective dose is more complex and error‐prone than in adults because various correction factors are used to convert the adult to the pediatric dose.^18^, ^19^ It is important to know the accurate radiation dose at pediatric CT performed at the lower tube voltages.

Our results show that there are no significant differences in radiation dose with each tube voltage among each phantom. In each phantom the newborn, 1 and 5 year‐old, the change from 120 to 100 kVp is decreased in the radiation dose (average decrease 11%, 7%, and 7%) and from 120 to 80 kVp decreased in the radiation dose (average decrease 12%, 19%, and 19%) while a constant noise level was maintained with TCM. Karmazyn et al.^20^ reported that in a 10 cm diameter circular cylinder phantom there was no significant change in the radiation dose when the tube voltage was decreased from 120 to 80 kVp. Also, in phantoms with a diameter of 20, 25, or 30 cm, the change from 120 to 100 kVp resulted in only a minimal increase in the dose (average increase 1%, 4%, and 6%, respectively) while a constant noise level was maintained without TCM. However, radiation dose of their results were increasing while using lower tube voltage. This may be attributable to our use of TCM because in this technique the tube current is automatically adjusted in the x and y planes (angular TCM), the z plane (z‐axis TCM), or both [three‐dimensional (3D) TCM] by inputting an appropriate noise value.^2^, ^21^ In the angular‐modulation techniques they automatically adjust the tube current for each projection angle to attenuation of the patients to minimize x‐rays in projection angle. In the z‐axis‐modulation technique, the system determines the tube current by using the patient localizer radiograph projection data and set of empirically determined noise projection coefficients by using reference technique. In earlier studies^22^, ^23^,3D TCM was on effective method for reducing the radiation dose delivered to patients and it could also reduce the radiation dose especially with respect to the anterior and posterior parts of the body. Therefore, at lower tube voltage settings the radiation dose may be minimally decreased in all phantoms.

When CT studies are performed in children, every effort must be made to select the optimal scanning protocols because they are more radiosensitive than adults and the radiation‐induced stochastic effects are prolonged. Knowing and understanding the ALARA (as low as reasonably achievable) concept is imperative for making informed decisions regarding linical CT, research protocols, and long‐term risk assessments.

The difference in the organ dose of low and standard tube voltage was minimal using pediatric phantoms. Shimonobo et al.^24^ reported that among the pediatric phantoms there was no statistically significant difference in the mean surface and center dose at 80, 100, and 120 kVp until image noise level was maintained. In our results, surface dose of skin and center dose of lungs had no significant difference among the different tube voltages. Especially under the 5‐year‐old children it may not be on influence during different tube voltages.

Efforts of the AAPM to refine CTDIvol as SSDE with patient diameter are fairly accurate, with 10–20% variability.^25^ In our study, the measured radiation dose was similar to the SSDE during lower tube voltage, it may be recommended to estimate SSDE on the basis of the conversion factors provided in AAPM Report 204.

Our study has some limitations. Firstly, we used anthropomorphic phantoms of newborn, 1‐year‐old and 5‐year‐old children to focus on routine and follow‐up studies in pediatric patients. As they grow older, their body size increases and the results may be different. Secondly, our studies were performed on a single CT scanner model, from a single manufacturer. The relationship among the tube voltages, image noise, radiation dose, and phantom size may depend to some degree on the CT scanner specifications that may vary among manufacturers. Thirdly, we performed one helical scan for the new born, 1 year‐old, and 5 year‐old phantom at each tube voltage. Lastly, we performed CT scan at one time for individual anthropomorphic phantoms.

## 5 CONCLUSIONS

Our anthropomorphic phantom study of the relationship between the radiation dose at different tube voltages in nonenhanced pediatric CT examinations suggests that the radiation dose at different low tube voltages is not significantly different when TCM is used.

## CONFLICT OF INTEREST

The authors declare no conflict of interest.

## Supporting information

Data S1: Measurement values with measurement portion for each organs in 80, 100, 120 kVp using anthropomorphic phantoms of a newborn, a one year old, and a 5 year‐old human. The measured portions are identified in Figs 5, 6, and 7.
