# Supplementary material for: Relationship between the radiation doses at nonenhanced CT studies using different tube voltages and automatic tube current modulation during anthropomorphic phantoms of young children
Source: J Appl Clin Med Phys. 2017 Oct 5;18(6):232–43. doi: 10.1002/acm2.12192 (PMC5689931; doi:10.1002/acm2.12192)
Supplement: Supplementary file 1 — Data S1: Measurement values with measurement portion for each organs in 80, 100, 120 kVp using anthropomorphic phantoms of a newborn, a one year old, and a 5 year‐old human. The measured portions are identified in Figs 5, 6, and 7. [file ACM2-18-232-s001.docx]

SUPPLEMENTAL MATERIALS

| Measurement portion | Organ (New-born phantom) | Measurement values (µGy) | | |
| --- | --- | --- | --- | --- |
|  |  | 80kVp | 100kVp | 120kVp |
| 1a | Bone marrow | 2017 | 2215 | 2301 |
| 1b | Bone marrow | 1803 | 2399 | 2640 |
| 1c | Bone marrow | 1998 | 1770 | 2380 |
| 1d | Bone marrow | 2105 | 2176 | 2114 |
| 1e | Bone marrow | 2060 | 2664 | 2710 |
| 1f | Bone marrow | 2316 | 2525 | 2700 |
| 1g | Bone marrow | 461 | 444 | 417 |
| 1h | Bone marrow | 1561 | 1786 | 1890 |
| 2a | Colon | 600 | 3600 | 2800 |
| 2b | Colon | 2635 | 2436 | 3190 |
| 2c | Colon | 2312 | 2749 | 2790 |
| 3a | Lung | 1906 | 2379 | 2120 |
| 3b | Lung | 2380 | 2485 | 2560 |
| 3c | Lung | 2386 | 2447 | 2320 |
| 3d | Lung | 2636 | 2430 | 2700 |
| 3e | Lung | 2518 | 2295 | 2820 |
| 3f | Lung | 2662 | 1970 | 3270 |
| 4a | Stomach | 2549 | 2620 | 2500 |
| 4b | Stomach | 2722 | 3610 | 3230 |
| 5a | Breast | 2348 | 2163 | 2820 |
| 5b | Breast | 2513 | 1917 | 3120 |
| 6a | Scapula | 2390 | 1948 | 2630 |
| 6b | Scapula | 2100 | 2303 | 2334 |
| 6c | Rib | 2421 | 2368 | 2530 |
| 6d | Rib | 2403 | 2782 | 2830 |
| 6e | Heart | 2593 | 2040 | 2840 |
| 6f | Adrenals | 2202 | 2155 | 2431 |
| 6g | Adrenals | 2647 | 2380 | 2450 |
| 6h | Rib | 2690 | 2990 | 1870 |
| 6j | Heart | 2391 | 3160 | 1820 |
| 6k | Rib | 2019 | 3030 | 1940 |
| 6l | Kidneys | 2137 | 2320 | 2310 |
| 6m | Kidneys | 2732 | 2882 | 2750 |
| 6n | Spleen | 2747 | 2824 | 2780 |
| 6o | Kidneys | 2007 | 2682 | 2830 |
| 6p | Kidneys | 2486 | 2097 | 2640 |
| 6q | Small intestine | 1969 | 2432 | 2780 |
| 6r | Pancreas | 2898 | 2796 | 3330 |
| 6s | Uterus | 2079 | 2418 | 2770 |
| 6t | Small intestine | 2938 | 2826 | 2790 |
| 6u | Gluteus maximus | 2264 | 2250 | 2630 |
| 6v | Gluteus maximus | 2453 | 2337 | 2990 |
| 6w | Lens | 208 | 156 | 112 |
| 6x | Lens | 368 | 76 | 272 |
| 6y | Lung | 2010 | 2427 | 1920 |
| 7a | Testicle | 2592 | 2531 | 2740 |
| 8a | Ovaries | 2063 | 2571 | 2560 |
| 8b | Ovaries | 2301 | 2393 | 2670 |
| 9a | Bladder | 2204 | 2539 | 3120 |
| 9b | Bladder | 1960 | 2070 | 2280 |
| 10a | Esophagus | 2056 | 2351 | 1856 |
| 10b | Esophagus | 2236 | 2482 | 2570 |
| 10c | Esophagus | 1842 | 1949 | 1818 |
| 11a | Liver | 2906 | 2290 | 2720 |
| 11b | Liver | 2399 | 2728 | 2760 |
| 11c | Liver | 2396 | 2938 | 2670 |
| 12a | Thyroid | 2801 | 2631 | 2820 |
| 12b | Thyroid | 2900 | 2740 | 3580 |
| 13a | Bone surface | 2763 | 1782 | 3450 |
| 13b | Bone surface | 2367 | 2072 | 2840 |
| 13c | Bone surface | 2171 | 2308 | 2880 |
| 13d | Bone surface | 2455 | 1925 | 2750 |
| 13e | Bone surface | 2065 | 2705 | 2580 |
| 13f | Bone surface | 2093 | 2469 | 2470 |
| 13g | Bone surface | 1301 | 2090 | 1341 |
| 14a | Brain | 86 | 160 | 125 |
| 14b | Brain | 28 | 144 | 75 |
| 14c | Brain | 45 | 154 | 155 |
| 14d | Brain | 45 | 127 | 106 |
| 14e | Brain | 306 | 134 | 234 |
| 14f | Brain | 229 | 158 | 153 |
| 14g | Brain | 88 | 216 | 43 |
| 15a | Salivary glands | 146 | 213 | 286 |
| 15b | Salivary glands | 321 | 53 | 292 |
| 15c | Salivary glands | 394 | 266 | 512 |
| 16a | Chest skin on left side | 2849 | 2510 | 2530 |
| 16b | Chest skin on right side | 2257 | 1871 | 2850 |
| 16c | Abdominal skin on anterior | 2271 | 3065 | 2910 |
| 16d | Chest skin on anterior | 2570 | 2493 | 2290 |
| 16e | Abdominal skin on right side | 2569 | 2413 | 2520 |
| 16f | Abdominal skin on left side | 2784 | 2352 | 2700 |
| 16g | Chest skin on posterior | 2291 | 2368 | 2240 |
| 16h | Abdominal skin on posterior | 1995 | 2447 | 2340 |

| Measurement portion | Organ (1-year-old phantom) | Measurement values (µGy) | | |
| --- | --- | --- | --- | --- |
|  |  | 80kVp | 100kVp | 120kVp |
| 1a | Bone marrow | 1543 | 1688 | 2206 |
| 1b | Bone marrow | 1674 | 2080 | 2252 |
| 1c | Bone marrow | 2520 | 2340 | 3590 |
| 1d | Bone marrow | 1740 | 3660 | 3000 |
| 1e | Bone marrow | 1990 | 2370 | 3050 |
| 1f | Bone marrow | 1930 | 3040 | 2690 |
| 1g | Bone marrow | 2310 | 3030 | 2880 |
| 1h | Bone marrow | 2115 | 2431 | 2773 |
| 2a | Colon | 2387 | 2895 | 3217 |
| 2b | Colon | 2164 | 2866 | 2660 |
| 2c | Colon | 1806 | 3214 | 2670 |
| 2d | Colon | 2110 | 2815 | 2690 |
| 3a | Lung | 2030 | 2097 | 2610 |
| 3b | Lung | 1917 | 2087 | 2490 |
| 3c | Lung | 2155 | 2638 | 2709 |
| 3d | Lung | 2174 | 2505 | 2879 |
| 3e | Lung | 2780 | 5830 | 4690 |
| 3f | Lung | 3240 | 3770 | 4390 |
| 3g | Lung | 3610 | 2470 | 4630 |
| 3h | Lung | 3180 | 2710 | 3880 |
| 4a | Stomach | 2920 | 3050 | 3800 |
| 4b | Stomach | 2790 | 4530 | 3290 |
| 5a | Breast | 2115 | 3270 | 2742 |
| 5b | Breast | 2310 | 2421 | 3120 |
| 6a | Extra-thoracic region | 2000 | 3150 | 3740 |
| 6b | Heart | 2580 | 3340 | 3770 |
| 6c | Heart | 2780 | 2440 | 4010 |
| 6d | Heart | 2850 | 3800 | 4230 |
| 6e | Heart | 3040 | 2920 | 5190 |
| 6f | Gallbladder | 2440 | 3220 | 3620 |
| 6g | Spleen | 2560 | 2750 | 4620 |
| 6h | Adrenals | 2370 | 3660 | 2620 |
| 6i | Adrenals | 2040 | 3290 | 2560 |
| 6j | Pancreas | 2120 | 3120 | 3350 |
| 6k | Kidneys | 2910 | 3220 | 2730 |
| 6l | Kidneys | 2260 | 3890 | 2770 |
| 6m | Spleen | 2510 | 3920 | 3780 |
| 6n | Pancreas | 2680 | 3259 | 3600 |
| 6o | Kidneys | 2238 | 2777 | 2570 |
| 6p | Kidneys | 2091 | 2694 | 2616 |
| 6q | Ileum | 2487 | 2841 | 3560 |
| 6r | Kidneys | 2318 | 2832 | 2900 |
| 6s | Kidneys | 2567 | 2815 | 3090 |
| 6t | Pancreas | 2711 | 3473 | 3400 |
| 6u | Uterus | 2200 | 3410 | 2470 |
| 6v | Lens | 3000 | 4100 | 2500 |
| 6w | Lens | 4200 | 3300 | 2100 |
| 7a | Testicle | 2710 | 4030 | 3440 |
| 8b | Ovaries | 2261 | 2774 | 3110 |
| 8b | Ovaries | 1930 | 3870 | 2060 |
| 9a | Bladder | 2460 | 3270 | 3190 |
| 9b | Bladder | 2200 | 3480 | 2890 |
| 10a | Esophagus | 1780 | 2088 | 2412 |
| 10b | Esophagus | 2246 | 2194 | 2428 |
| 10c | Esophagus | 1990 | 2730 | 3050 |
| 10d | Esophagus | 3110 | 2440 | 4210 |
| 10e | Esophagus | 2170 | 2930 | 4870 |
| 11a | Liver | 2350 | 2320 | 4530 |
| 11b | Liver | 2290 | 2650 | 3800 |
| 11c | Liver | 1830 | 4040 | 3800 |
| 11d | Liver | 2320 | 3120 | 4560 |
| 11e | Liver | 3020 | 3170 | 3230 |
| 11f | Liver | 2593 | 3184 | 2960 |
| 12a | Thyroid | 1693 | 2185 | 2619 |
| 12b | Thyroid | 7100 | 3500 | 9000 |
| 13a | Bone surface | 2259 | 1851 | 2387 |
| 13b | Bone surface | 1818 | 2326 | 2381 |
| 13c | Bone surface | 2680 | 2920 | 2770 |
| 14a | Brain | 33 | 71 | 96 |
| 14b | Brain | 67 | 80 | 119 |
| 14c | Brain | 119 | 156 | 160 |
| 14d | Brain | 88 | 153 | 158 |
| 15a | Salivary glands | 810 | 530 | 1030 |
| 15b | Salivary glands | 560 | 580 | 1050 |
| 15c | Salivary glands | 420 | 900 | 820 |
| 16a | Chest skin on anterior | 5400 | 900 | 10000 |
| 16b | Chest skin on posterior | 4200 | 4700 | 7800 |
| 16c | Chest skin on right side | 4940 | 4500 | 7100 |
| 16d | Chest skin on left side | 2500 | 3480 | 7200 |
| 16e | Abdominal skin on anterior | 3400 | 3000 | 6800 |
| 16f | Abdominal skin on posterior | 3100 | 6100 | 9100 |
| 16g | Abdominal skin on right side | 2492 | 2656 | 2898 |
| 16h | Abdominal skin on left side | 2020 | 2349 | 2644 |

| Measurement portion | Organ (5-year-old phantom) | Measurement values (µGy) | | |
| --- | --- | --- | --- | --- |
|  |  | 80kVp | 100kVp | 120kVp |
| 1a | Bone marrow | 497 | 428 | 857 |
| 1b | Bone marrow | 1850 | 2230 | 2220 |
| 1c | Bone marrow | 1640 | 2420 | 2780 |
| 1d | Bone marrow | 2560 | 1740 | 3210 |
| 1e | Bone marrow | 1760 | 2070 | 3100 |
| 1f | Bone marrow | 1280 | 3410 | 3960 |
| 1g | Bone marrow | 1870 | 1100 | 6010 |
| 1h | Bone marrow | 2920 | 2170 | 4210 |
| 2a | Colon | 2630 | 2640 | 3980 |
| 2b | Colon | 2700 | 3200 | 3650 |
| 2c | Colon | 2380 | 2720 | 3560 |
| 3a | Lung | 1008 | 2040 | 3860 |
| 3b | Lung | 2151 | 2080 | 2850 |
| 3c | Lung | 2750 | 2110 | 3970 |
| 3d | Lung | 2690 | 2800 | 3460 |
| 3e | Lung | 2820 | 2280 | 4370 |
| 3f | Lung | 3230 | 3480 | 4250 |
| 3g | Lung | 2300 | 4570 | 3140 |
| 3h | Lung | 3410 | 2680 | 4420 |
| 4a | Stomach | 3680 | 1680 | 6390 |
| 4b | Stomach | 3860 | 2650 | 6770 |
| 5a | Breast | 2290 | 3030 | 3890 |
| 5b | Breast | 2630 | 2870 | 3560 |
| 6a | Extra-thoracic region | 2339 | 790 | 3170 |
| 6b | Extra-thoracic region | 1901 | 2460 | 3740 |
| 6c | Heart | 2950 | 4500 | 3120 |
| 6d | Heart | 600 | 1300 | 10000 |
| 6e | Heart | 3010 | 2840 | 4280 |
| 6f | Heart | 3380 | 3670 | 4610 |
| 6g | Heart | 1750 | 1990 | 10170 |
| 6h | Gallbladder | 3070 | 3270 | 4450 |
| 6i | Spleen | 2530 | 3320 | 5230 |
| 6j | Adrenals | 2730 | 3020 | 5060 |
| 6k | Adrenals | 1790 | 4260 | 3580 |
| 6l | Pancreas | 2500 | 800 | 5530 |
| 6m | Kidneys | 1790 | 3360 | 3290 |
| 6n | Kidneys | 2680 | 3730 | 3760 |
| 6o | Spleen | 3760 | 1870 | 6650 |
| 6p | Small intestine | 2930 | 2340 | 5660 |
| 6q | Pancreas | 3910 | 4170 | 4090 |
| 6r | Kidneys | 2500 | 3130 | 3730 |
| 6s | Kidneys | 2550 | 2970 | 3670 |
| 6t | Kidneys | 2790 | 3740 | 3810 |
| 6u | Kidneys | 3180 | 3390 | 2920 |
| 6v | Pancreas | 3440 | 4050 | 4570 |
| 6w | Uterus | 2160 | 3630 | 3140 |
| 6x | Lens | 3800 | 2200 | 300 |
| 6y | Lens | 3000 | 1400 | 1000 |
| 7a | Testicle | 4020 | 2560 | 6580 |
| 8a | Ovaries | 2690 | 2970 | 4120 |
| 8b | Ovaries | 1990 | 3690 | 4180 |
| 9a | Bladder | 3020 | 3020 | 4710 |
| 9b | Bladder | 2940 | 3720 | 4340 |
| 10a | Esophagus | 2777 | 2030 | 3890 |
| 10b | Esophagus | 2521 | 2060 | 2260 |
| 10c | Esophagus | 2110 | 2170 | 2830 |
| 10d | Esophagus | 3150 | 3150 | 2830 |
| 10e | Esophagus | 3290 | 2670 | 4170 |
| 11a | Liver | 3130 | 3160 | 3590 |
| 11b | Liver | 3220 | 2260 | 3950 |
| 11c | Liver | 2780 | 3410 | 4460 |
| 11d | Liver | 4230 | 3180 | 4690 |
| 11e | Liver | 2050 | 5370 | 3270 |
| 11f | Liver | 2970 | 2760 | 4980 |
| 12a | Thyroid | 2515 | 2840 | 2990 |
| 12b | Thyroid | 2400 | 5300 | 2400 |
| 13a | Bone surface | 83 | 142 | 112 |
| 13b | Bone surface | 1460 | 3090 | 3050 |
| 13c | Bone surface | 2170 | 1940 | 4370 |
| 14a | Brain | 50 | 55 | 44 |
| 14b | Brain | 36 | 56 | 79 |
| 14c | Brain | 74 | 69 | 178 |
| 15a | Salivary glands | 210 | 120 | 540 |
| 15b | Salivary glands | 270 | 170 | 250 |
| 15c | Salivary glands | 50 | 180 | 440 |
| 16a | Chest skin on anterior | 2100 | 2400 | 5000 |
| 16b | Chest skin on posterior | 2200 | 4700 | 3800 |
| 16c | Chest skin on right side | 1200 | 3300 | 4100 |
| 16d | Chest skin on left side | 1000 | 3000 | 5300 |
| 16e | Abdominal skin on anterior | 500 | 1500 | 6500 |
| 16f | Abdominal skin on posterior | 500 | 3300 | 5100 |
| 16g | Abdominal skin on right side | 4060 | 3810 | 4370 |
| 16h | Abdominal skin on left side | 3322 | 2770 | 4010 |
