# Supplementary material for: Relationship between the radiation doses at nonenhanced CT studies using different tube voltages and automatic tube current modulation during anthropomorphic phantoms of young children
Source: J Appl Clin Med Phys. 2017 Oct 5;18(6):232–43. doi: 10.1002/acm2.12192 (PMC5689931; doi:10.1002/acm2.12192)
Supplement: Supplementary file 2 [file ACM2-18-232-s002.docx]

**Data S1.** Measurement values with measurement portion for each organs in 80, 100, 120 kVp using anthropomorphic phantoms of a newborn, a one year old, and a 5 year-old human. The measured portions are identified in Figs 5, 6, and 7.
